# Association of Glomerular Filtration Rate with High-Sensitivity Cardiac Troponin T in a Community-Based Population Study in Beijing

**DOI:** 10.1371/journal.pone.0038218

**Published:** 2012-05-31

**Authors:** Fan Wang, Ping Ye, Leiming Luo, Ruyi Xu, Yongyi Bai, Hongmei Wu

**Affiliations:** Department of Geriatric Cardiology, Chinese PLA General Hospital, Beijing, China; California Pacific Medicial Center Research Institute, United States of America

## Abstract

**Background:**

Reduced renal function is an independent risk factor for cardiovascular disease mortality, and persistently elevated cardiac troponin T (cTnT) is frequently observed in patients with end-stage renal disease. In the general population the relationship between renal function and cTnT levels may not be clear because of the low sensitivity of the assay. In this study, we investigated the level of cTnT using a highly sensitive assay (hs-cTnT) and evaluated the association of estimated glomerular filtration rate (eGFR) with detectable hs-cTnT levels in a community-based population.

**Methods:**

The serum hs-cTnT levels were measured in 1365 community dwelling population aged ≥45 years in Beijing, China. eGFR was determined by the Chinese modifying modification of diet in renal disease (C-MDRD) equation.

**Results:**

With the highly sensitive assay, cTnT levels were detectable (≥3pg/mL) in 744 subjects (54.5%). The result showed that eGFR was associated with Log hs-cTnT (r = −0.14, *P*<0.001). After adjustment for the high predicted Framingham Coronary Heart Disease (CHD) risk (10-year risk >20%) and other prognostic indicators, moderate to severe reduced eGFR was independently associated with detectable hs-cTnT, whereas normal to mildly reduced eGFR was not independently associated with detectable hs-cTnT. In addition, after adjustment for other risk factors, the high predicted Framingham CHD risk was associated with detectable hs-cTnT in the subjects with different quartile levels of eGFR.

**Conclusion:**

The levels of hs-cTnT are detectable in a community-based Chinese population and low eGFR is associated with detectable hs-cTnT. Moreover, eGFR and high predicted Framingham CHD risk are associated with detectable hs-cTnT in subjects with moderate-to-severe reduced renal function.

## Introduction

A reduced glomerular filtration rate is an independent risk factor for cardiovascular disease mortality [1]–[2]. Meanwhile, several large-scale prospective clinical trials have shown that the prevalence of adverse cardiovascular disease events is increased among patients with end-stage renal disease [3]–[4]. Also, persistently elevated levels of cardiac biomarkers are frequently observed in these patients [5]–[6], such as cardiac troponin T (cTnT), as a highly sensitive and specific marker of myocardial damage. In the asymptomatic general population, however, the relationship between renal function and myocardial injury is not clear. The clinical use of serum levels of cardiac biomarkers has limitations because of the low sensitivity of the assay systems [7]. The prevalence of detectable concentrations of cTnT in the general population is approximately 0.7% with the use of conventional assays [8].

Recently, a highly sensitive cardiac troponin T (hs-cTnT) assay has become commercially available permitting measurement of concentrations that are lower by a factor of 10 than those measurable with conventional assays [9]. Indeed, as recommended in the recent guideline for biomarker evaluation, optimal precision (the coefficient of variation (CV) at the 99th percentile upper reference limit for assays should be defined as ≤10%) and reliable precision allows for sensitive assays [10]. cTnT with a highly sensitive assay meets the recommendation and provides a sensitive assay to study the relationship between renal function and myocardial damage or subclinical myocardial damage in asymptomatic subjects with normal-to-mild reduced renal function.

The Framingham Coronary Heart Disease (CHD) risk prediction score is calculated by the individual variables that constitute the risk score, including sex, age, low-density lipoprotein (LDL)-cholesterol, SBP, DBP, history of diabetes mellitus (DM), and current smoking [11]. Our previous study had confirmed that the Framingham CHD risk prediction score is independently and positively associated with detectable hs-cTnT [12]. In this study we investigated the relationship between estimated glomerular filtration rate (eGFR) and detectable hs-cTnT, and the role of the high predicted Framingham CHD risk (10-year risk >20%) in a community-based population in Beijing, China.

## Methods

The study protocol was approved by the Ethics Committee of the Chinese People's Liberation Army (PLA) General Hospital (Beijing, China). Each participant provided written informed consent to be included in the study.

### Study population

This was a community-based cross-sectional study of people living in the Pingguoyuan area of Shijingshan district, a metropolitan area in Beijing, China. All participants were permanent residents of Han origin, aged ≥45 years, who were recruited to the study after a routine health check-up from September 2007 to January 2009. Subjects with bedridden status, mental illness, malignant tumors and severe systemic diseases were excluded from the analysis. Initially, 1,601 participants were included. Among them, 1,503 subjects provided blood samples for the testing of cardiac biomarkers. Of those, 6 subjects had missing data, such as without serum lipids, blood glucose or serum creatinine. Therefore, a total of 1,497 participants with complete data were eligible for assessment. Eventually, after excluding 132 participants for overt vardiovascular disease, 1365 participants formed the present study.

### Questionnaire and anthropometric measurements

Information about smoking status, medication use, a history of hypertension, DM, and coronary heart disease was obtained by self-reporting, standardized questionnaires. This was administered using a face-to-face counseling method. The investigation was completed by physicians in the Department of Geriatric Cardiology of the People's Liberation Army General Hospital who were trained by the research team.

Height, weight and circumferences of the waist and hip were measured. The body mass index (BMI) and waist-to-hip ratio (WHR) were calculated. BMI was calculated as weight in kilograms divided by the height in meters squared (kg/m^2^). WHR was calculated as waist circumference (WC) divided by hip circumference. The measurement of blood pressure was done using a calibrated desktop sphygmomanometer (Yuyue, Armamentarium Limited Company, Jiangsu, China) after participants had been in the supine position for ≥5 min [13]. Blood pressure was measured thrice consecutively, with ≥1 min between measurements. The mean value of blood pressure was used for the statistical analysis.

### Biomarker measurements

Blood samples were collected in tubes containing separating gel after overnight fasting and maintained at 4°C for ≤2 h before being centrifuged at 1200× *g* for 15 min. Serum aliquots were frozen at −80°C until assays were carried out.

Concentrations of fasting glucose, total cholesterol, triglyceride, high-density lipoprotein (HDL)-cholesterol, LDL-cholesterol, uric acid and homocysteine were determined using the enzymatic assays (Roche Diagnostics GmbH, Mannheim, Germany) on an autoanalyzer (Roche Diagnostics, Indianapolis, IN, USA). Concentrations of hs-cTnT were determined using a Elecsys Troponin T highly sensitivity assay (Roche Diagnostics GmbH, Mannheim, Germany) by an electrochemiluminescence immunoassay method on a Modular Analytics E170 Autoanalyzer (Roche Diagnostics). Given enhanced sensitivity, this assay was reported in units of picograms per milliliter (pg/mL) with an interassay coefficient of variation of 8% at 10 pg/mL and 2.5% at 100 pg/mL [14]. The lower detection limit of the hs-cTnT assay was 3 pg/mL (according to manufacturer's information), which was used as the cut-off point in the present analysis; hs-cTnT levels less than 3 pg/mL were considered undetectable (<3.0 pg/mL). The 99th percentile for a reference population has been reported to be 13.3 pg/mL [10]. Concentrations of the N-terminal prohormone of brain natriuretic peptide (NT-proBNP) were determined with an electrochemiluminescence immunoassay (Roche Diagnostics GmbH) using a Roche analyzer. It had a measurement range of 5–35000 pg/mL. According to manufacturer's information, the lower limit of detection was 5 pg/mL [15]. Concentrations of high-sensitivity C-reactive protein (hs-CRP) were determined by an immunoturbidimetric assay (Siemens Healthcare Diagnostics, Los Angeles, CA, USA) using a Dimension RxL Max analyzer (Siemens Healthcare Diagnostics). Concentrations of serum creatinine were measured by an enzymatic assay (Roche Diagnostics GmbH) on a Hitachi 7600 Autoanalyser (Hitachi, Tokyo, Japan). All testing was undertaken by well-trained personnel blinded to clinical data in the Department of Biochemistry of Chinese PLA General Hospital.

### Definition of variables

Cigarette smoking was assessed by asking each individual whether he/she was a current smoker. A subject was considered to have hypertension if (i) systolic blood pressure (SBP) ≥140 mmHg; and/or (ii) diastolic blood pressure (DBP) ≥90 mmHg; and/or (iii) the subject was taking an antihypertensive drug [16].

All participants without a history of DM were given a standard 75 g oral glucose tolerance test (OGTT). Results showed that 1286 participants had the OGTT. Fasting venous blood was collected from participants with a history of DM to measure blood glucose. A subject was considered to have DM if (i) fasting venous blood glucose ≥7.1 mmol/L; (ii) 2 h venous blood glucose ≥11.1 mmol/L; or (iii) the subject was taking a hypoglycemic drug or insulin [17].

Renal function was evaluated by eGFR. Creatinine level was standardized using a calibration equation: Jaffe's kinetic method Scr (mg/dL) = 0.795×[enzymatic method Scr (mg/dL)]+0.29 [18]. eGFR was calculated using the Chinese modifying modification of diet in renal disease (C-MDRD) equation [19]: eGFR (mL/min/1.73 m^2^) = 175×standard creatinine (mg/dL)^−1.234^×age (year)^−0.179^×(0.79 if female).

### Calculation of predicted risk

The Framingham CHD risk prediction score incorporates sex, age, LDL-cholesterol, SBP, DBP, history of DM, and current smoking [11]. The levels of 10-year risk for the CHD events were categorized as: low (10-year risk <10%); moderate (10-year risk 10% to 20%); or high (10-year risk >20%) [20]. In the present analysis, the high predicted Framingham CHD risk (10-year risk >20%) was used as the cut-off point.

### Statistical analyses

Characteristics are reported as percentages for categorical variables and means (±SD) or median (with interquartile range) for continuous variables. The hs-cTnT levels were presented both as a continuous variable (after natural logarithmic transformation) and as a categorical variable when appropriate. The hs-cTnT was classified as undetectable (<3 pg/mL), and detectable (≥3 pg/mL). eGFR levels were categorized as: quartile 1 (≥97.02 mL/min/1.73 m^2^), quartile 2 (97.01 to 87.76 mL/min/1.73 m^2^), quartile 3 (87.75 to 78.97 ml/min/1.73 m^2^), and quartile 4 (≤78.96 mL/min/1.73 m^2^). The quartile 1 to 3 levels of eGFR were defined as the normal to mild reduced renal function; the quartile 4 level of eGFR was defined as the moderate to severe reduced renal function. Statistical comparison of groups was undertaken by one-way ANOVA (continuous variables) or chi-square tests (categorical variables).

In order to evaluate the association between eGFR and hs-cTnT as a continuous variable (natural logarithm transformed), the Pearson's correlation for continue variables or the Spearman's correlation for categorical variables was used in univariable analyses, and multivariable linear regression analysis was performed requiring a variable with a probability value of ≤0.10 to be entered and <0.05 to remain in the model after adjusting for several potential confounders (covariates). In the analysis, undetectable hs-cTnT levels (<3 pg/mL) were considered 1.5 pg/mL [21].

In addition, to better understand the association between different quartile levels of eGFR and detectable hs-cTnT, logistic regression models were used. Forward stepwise multivariable logistic regression was performed to obtain the odds ratios (OR) and 95% confidence intervals (CI) requiring a variable with a probability value of ≤0.10 to be entered and <0.05 to remain in the model and the quartile 1 level of eGFR was used as the reference. Regression models were adjusted for high predicted Framingham CHD risk (10-year risk ≥20%) (model 1) and for model 1 plus levels of BMI, WC, and WHR (model 2). Model 3 was adjusted for model 2 plus levels of fasting glucose, uric acid, hs-CRP, homocysteine and NT-proBNP.

Furthermore, in order to investigate the role of high predicted Framingham CHD risk in the relationship between eGFR and hs-cTnT, we evaluate the association between high predicted Framingham CHD risk (10-year risk ≥20%) and detectable hs-cTnT in subjects with different quartile levels of eGFR, forward stepwise multivariable logistic regression was repeatedly used, a variable with a probability value of ≤0.10 to be entered and <0.05 to remain in the model. Regression models were adjusted for BMI, WC, and WHR (model 1) and for model 1 plus levels of fasting glucose, uric acid, hs-CRP, homocysteine and NT-proBNP (model 2).

All data entry and management were undertaken on an Excel spreadsheet and were then analyzed by the SPSS statistical package (version 16.0; SPSS Inc., Chicago, IL, USA). A 2-sided value of P<0.05 was considered significant.

## Results

### Clinical characteristics of participants

A total of 1365 subjects were included in the analysis. There were 578 males (42.3%) and 787 females (57.7%). The age range was 45 to 96 years old (mean, 62.36±9.82 years). Of these, there were 351 current smokers (25.7%), 239 DM patients (17.5%), 605 hypertensive individuals (44.3%). There were 28 subjects with eGFR ≤60 ml/min/1.73 m^2^ (1.89%).

Table 1 shows the clinical characteristics of the study population. Participants were divided into four groups based on the level of the quartile of eGFR (≥97.02, 97.01–87.76, 87.75–78.97, ≤78.96 mL/min/1.73 m^2^). Compared to the quartile 1 of eGFR, age, the percentage of hypertension and diabetes, the level of SBP, total cholesterol, uric acid, homocysteine, hs-CRP, log NT-proBNP, log hs-cTnT and high predicted Framingham CHD risk score (10-year risk ≥20%) in the quartile 4 of eGFR were higher (*P*<0.05).

**Table 1:** The clinical characteristics of study participants.

	Overall	Quartile 1	Quartile 2	Quartile 3	Quartile 4
	(n = 1365)	≥97.02	97.01-87.76	87.75-78.97	≤78.96
Characteristic		(n = 342)	(n = 341)	(n = 341)	(n = 341)
eGFR (ml/min/1.73 m^2^)	87.76(78.98,97.03)	107.36(100.45,115.98)	91.74(89.47,93.99)*	83.47(81.14,85.68)*	72.04(67.24,75.99)*
Age (years)	62.36±9.82	60.51±9.64	59.05±8.93	62.04±9.54	67.84±8.83#
male sex [n (%)]	578(42.3%)	138(40.4%)	146(42.8%)	143(41.3%)	151(44.3%)
BMI (kg/m^2^)	25.61±3.51	25.53±3.56	25.65±3.68	25.54±3.40	25.73±3.41
WC (cm)	86.90±9.78	86.29±9.92	86.85±10.27	86.59±9.70	87.90±9.17
Waist-hip ratio	0.87±0.06	0.87±0.06	0.87±0.06	0.87±0.07	0.88±0.06
Current smoking [n (%)]	351(25.7%)	91(26.6%)	80(23.5%)	85(24.9%)	95(27.9%)
Hypertension [n (%)]	605(44.3%)	143(41.8%)	135(39.6%)	158(46.3%)*	169(49.6%)*
Diabetes mellitus [n (%)]	239(17.5%)	78(22.8%)	53(15.5%)*	52(15.2%)*	56(16.4%)*
Systolic BP (mm Hg)	130.79±18.20	129.00±18.29	128.77±18.34	132.07±17.18*	133.31±18.60*
Diastolic BP (mm Hg)	76.86±10.71	77.51±10.84	77.38±10.66	76.52±9.81	76.04±11.45
Total cholesterol (mmol/L)	5.07±0.91	4.93±0.90	5.09±0.87	5.10±0.93	5.18±0.92#
Triglyceride (mmol/L)	1.81±1.22	1.78±1.01	1.85±1.29	1.80±1.37	1.81±1.21
HDL cholesterol (mmol/L)	1.40±0.36	1.41±0.39	1.41±0.36	1.43±0.35	1.37±0.35
LDL cholesterol (mmol/L)	2.99±0.70	2.96±0.66	2.97±0.70	3.01±0.73	3.01±0.72
Fasting glucose (mmol/L)	5.42±1.68	5.56±2.10	5.48±1.43	5.39±1.72*	5.28±1.37*
Uric acid (µmol/L)	291.31±73.10	269.88±71.47	280.42±65.60#	297.31±72.78#	317.71±73.45#
Homocysteine (µmol/L)	19.16±8.56	17.69±7.33	18.05±7.61	18.79±8.58	22.12±9.81#
hs-CRP (mg/dL)	0.22(0.14,0.34)	0.20(0.14,0.33)	0.21(0.12,0.33)	0.23(0.13,0.36)	0.25(0.16,0.36)#
Log NT-proBNP (pg/mL)	3.72(2.95,4.39)	3.54(2.86,4.22)	3.48(2.67,4.21)	3.72(2.94,4.32)#	4.14(3.42,4.85)#
Log hs-cTnT (pg/mL)	1.27(1.10,2.08)	1.10(1.10,1.94)	1.10(1.10,1.91)	1.26(1.10,2.13)#	1.67(1.10,2.31)#
Framingham risk score, %	9.00(6.00,15.00)	9.00(5.00,14.00)	8.00(5.00,13.00)	9.00(6.00,14.00)	11.00(7.00,18.00)#
Framingham risk score ≥20, no. (%)	202(14.8%)	48(14.0%)	35(10.34%)	46(13.5%)	72(21.1%)#

**Note**: Characteristics are reported as percentages for categorical variables and means (±SD) or median (with interquartile range) for continuous variables. The study participants were divided into four groups based on the level of the quartile of eGFR (≥97.02, 97.01–87.76, 87.75–78.97, ≤78.96 mL/min/1.73 m^2^). Categorical variables are presented as counts and percentages. The values outside the parentheses are the number of subjects, and the values inside the parentheses are prevalence.

The quartile 1 level of eGFR was used as the reference and the quartile 2,3,4 vs the quartile 1, respectively.

* <0.05 vs Quartile 1,

# <0.01 vs Quartile 1.

eGFR, estimated glomerular filtration rate; hs-cTnT denotes high-sensitivity cardiac troponin T; BMI, bodymass index; WC, waist circumference; BP, blood pressure; HDL, high-density lipoprotein; LDL, low-density lipoprotein; hs-CRP, high-sensitivity C-reactive protein; NT-proBNP, N-terminal pro B-type.

### Concentration and Distribution of hs-cTnT

The range of detectable hs-cTnT concentrations was 3.03–176.40 pg/mL with a median value of 7.45 pg/mL (quartile 1 to quartile 3: 4.84–12.02 pg/mL) in this community-based population. Among 1365 participants, 621 subjects (45.5%) had undetectable hs-cTnT (<3 pg/mL), 580 subjects (42.5%) had hs-cTnT concentration 3–13.2 pg/mL, and 164 subjects (12.0%) had hs-cTnT concentration ≥13.3 pg/mL.

### The association between eGFR and hs-cTnT

The association between eGFR and hs-cTnT as a continuous variable (natural logarithm transformed) was presented in table 2. The results of Pearson's correlation showed that eGFR had a negative relationship with log hs-cTnT (*r* = −0.143; *P*<0.001). In multivariable linear regression analysis, eGFR was negatively and independently associated with hs-cTnT levels. In addition, the male, older age, NT-proBNP and fasting glucose were positively and independently associated with hs-cTnT levels.

**Table 2:** Pearson's correlation and Multiple linear regression analysis for the association between eGFR and the hs-cTnT levels.

Characteristic	Univariable		Multivariable	
	r	P Value	β	P Value
Age, yr	0.213	<0.001	0.112	<0.001
Male sex	0.240	<0.001	0.200	<0.001
Hypertension	0.135	<0.001	…	…
Diabetes mellitus	0.121	<0.001	…	…
Current smoking	0.085	0.002	…	…
BMI, kg/m^2^	0.022	0.409	…	…
Systolic BP, mm Hg	0.078	0.004	…	…
Diastolic BP, mm Hg	−0.034	0.208	…	…
Total cholesterol, mmol/L	−0.056	0.039	…	…
Triglyceride, mmol/L	−0.005	0.853	…	…
HDL cholesterol, mmol/L	−0.110	<0.001	…	…
LDL cholesterol, mmol/L	0.023	0.401	…	…
Fasting glucose, mmol/L	0.067	0.013	0.098	<0.001
hs-CRP, mg/L	0.019	0.472	…	…
Uric acid, µmol/L	0.136	<0.001	…	…
Homocysteine, µmol/L	0.194	<0.001	…	…
NT-proBNP, pg/mL	0.116	<0.001	0.169	<0.001
eGFR, mL/min/1.73 m^2^	−0.143	<0.001	−0.069	0.009

**Note**: High-sensitivity cardiac troponin T levels were natural logarithm transformed. BMI, body-mass index; BP, blood pressure; HDL, high-density lipoprotein; LDL, low-density lipoprotein; hs-CRP, high-sensitivity C-reactive protein; NT-proBNP, N-terminal pro B-type natriuretic peptide; eGFR, estimated glomerular filtration rate.

The relationship between different quartile levels of eGFR and detectable hs-cTnT is shown in Tables 3. A stepwise logistic regression model was performed and the quartile 1 level of eGFR was used as the reference. In the univariate model, all quartile levels of eGFR were associated with detectable hs-cTnT. After adjusted for high predicted Framingham CHD risk (10-year risk ≥20%) and/or other predicted factors, however, only quartile 4 of eGFR was independently associated with detectable hs-cTnT. eGFR in quartile 2 and 3 were not independently associated with detectable hs-cTnT in the adjusted models (Model 1, 2 and 3).

**Table 3:** Association between eGFR and detectable hs-cTnT.

	Quartile 1	Quartile 2	Quartile 3	Quartile 4
	≥97.02	97.01-87.76	87.75-78.97	≤78.96
Univariable model				
Odds ratio	1	1.016	1.573	2.164
95% CI	Reference	1.002–1.019	1.208–2.049	1.610–2.909
P		0.020	0.001	<0.001
Model 1				
Odds ratio	1	1.036	1.261	2.069
95% CI	Reference	0.774–1.386	0.942–1.689	1.531–2.796
P		0.813	0.119	<0.001
Model 2				
Odds ratio	1	1.019	1.175	1.950
95% CI	Reference	0.753–1.380	0.869–1.590	1.427–2.663
P		0.903	0.294	<0.001
Model 3				
Odds ratio	1	1.016	1.080	1.500
95% CI	Reference	0.746–1.382	0.793–1.471	1.078–2.086
P		0.922	0.625	0.016

**Note:**

Model 1: Adjusted for the high predicted Framingham CHD risk (10-year risk >20%).

Model 2: Adjusted for model 1 plus levels of body mass index; waist circumference and waist-hip ratio.

Model 3: Adjusted for model 2 plus levels of fasting glucose, uric acid, high-sensitivity C-reactive protein, homocysteine and NT-proBNP.

Tables 4 shows the relationship between the high predicted Framingham CHD risk (10-year risk ≥20%) and hs-cTnT value as a categorical variable (detectable or undetectable) in subjects with different quartile levels of eGFR. The results showed that the high Framingham CHD risk score was independently associated with detectable hs-cTnT in adjusted models (Modle 1, 2). Furthermore, the OR increased with decreasing quartile levels of eGFR (quartiles 1 to 3), whereas the OR decreased abruptly in the quartile 4 of eGFR.

**Table 4:** Association between the high predicted Framingham CHD risk (10-year risk >20%) and detectable hs-cTnT in different quartile level of eGFR.

	Quartile 1	Quartile 2	Quartile 3	Quartile 4
	≥97.02	97.01-87.76	87.75-78.97	≤78.96
Univariable model				
Odds ratio	3.487	3.614	4.779	1.873
95% CI	1.822–6.673	1.791–7.292	2.133–10.706	1.0515–3.336
P	<0.001	<0.001	<0.001	0.033
Multivariable model 1^a^				
Odds ratio	3.174	3.772	4.180	1.860
95% CI	1.634–6.164	1.639–8.685	2.009–8.698	1.372–2.533
P	0.001	0.002	<0.001	<0.001
Multivariable model 2^b^				
Odds ratio	2.810	3.296	3.750	1.407
95% CI	1.397–5.654	1.311–8.284	1.753–8.020	1.131–1.751
P	0.004	0.011	0.001	0.002

**Note:**

a Adjusted for body mass index; waist circumference and waist-hip ratio.

b Adjusted for model 1 plus levels of fasting glucose, uric acid, high-sensitivity C-reactive protein, homocysteine and NT-proBNP.

## Discussion

In this study, we demonstrated for the first time that, moderate-to-severe reduced eGFR (the level of quartile 4 of eGFR) was independently and negatively associated with detectable hs-cTnT. In contrast, normal-to-mild reduced eGFR (the level of quartile 1 to 3 of eGFR) was not independently associated with detectable hs-cTnT. Moreover, the high predicted Framingham CHD risk (10-year risk ≥20%) was independently and positively associated with detectable hs-cTnT in different quartile levels of eGFR. These results indicate that high predicted Framingham CHD risk maybe play a dominant role to affect the level of hs-cTnT in normal-to-mild reduced renal function, however, high predicted Framingham CHD risk and eGFR were conjunctly associated with detectable hs-cTnT in moderate-to-severe reduced renal function.

The mechanisms responsible for the release of very low levels of cTnT in the general population could include subclinical myocardial damage [22], inflammatory processes [23], reduced renal clearance and so on. Of there, subclinical myocardial damage may be the principal cause. The cTnT is an extremely sensitive and specific biomarker of myocardial necrosis [24]. Normally, the majority of troponin exists as a tripartite complex of C, I, and T components that are bound to actin filaments, and the remainder is free in the cytoplasm. When cardiomyocyte damage occurs, the cytoplasmic pool of troponin is released first and followed by a more protracted release from stores bound to deteriorate myofilaments [25]. Thus, cTnT is detetable with highly sensitive assay in subclinical myocardial damage, which is even in slight injury. However, few data are available for evaluating the prevalence of cTnT elevation in a large and representative sample of the general population because of the low sensitivity of the conventional assay system [7]. With the use of this highly sensitive assay, identification of subclinical myocardial damage is improved. In the present study, we found that circulating hs-cTnT levels were detectable in 54.5% of subjects and ≈12.0% of subjects had hs-cTnT concentrations ≥13.3 pg/mL. The results are similar to other studies [26], [27].

The interaction between the heart and kidney has been explored for a long time. Several studies have documented that patients with a progressive decrease in eGFR are at a higher risk of cardiovascular disease than the general population, and show a higher prevalence of cardiovascular mortality [28]–[30]. Levels of cTnT are frequently elevated in the absence of acute coronary occlusion among patients with renal dysfunction, specifically in 30–75% of end-stage renal disease patients [31]–[32]. However, the pathophysiologic mechanisms causing random increases in cTnT levels in patients with renal dysfunction or dialysis are unclear. Several reasons may help to explain this correlation. Firstly, with reduced of renal excretion, increase of volume load [33] or accumulation of a certain nephrotoxin *per se* [34] would lead to ischemia or damage to the myocardium. Secondly, troponin is usually believed to be cleared by the reticuloendothelial system given the relatively large molecular size of troponin [35]. However, recent evidence demonstrates that troponin T can be fragmented into molecules small enough to be excreted by the kidney; this may explain the high prevalence of troponin elevation in patients with severe renal failure [36]. Thirdly, with decreased renal function, the cardiovascular risk is increased and the possibility of subclinical myocardial damage raised [37]. In the present study, after stepwise adjustment for high predicted Framingham CHD risk and other cardiovascular prognostic indicators, such as BMI, NT-proBNP, hs-CRP, homocysteine and so on, normal-to-mild reduced levels of eGFR were not independently associated with detectable hs-cTnT. In contrast, moderate-to-severe reduced levels of eGFR were independently and negatively associated with detectable hs-cTnT. The results implied that other factors may have an effect on detectable hs-cTnT in normal-to-mild reduced renal function. While, high predicted Framingham CHD risk (representing traditional cardiovascular risk factors) perhaps is the important “other factors”.

Then in order to investigate the role of the predicted Framingham CHD risk in the relationship between eGFR and hs-cTnT, we evaluate the association between high predicted Framingham CHD risk (10-year risk ≥20%) and detectable hs-cTnT in subjects with different quartile levels of eGFR. The results showed that the high predicted Framingham CHD risk was independently associated with detectable hs-cTnT in different levels of eGFR in adjusted models. Furthermore, the OR for high predicted CHD risk increased with decreasing quartile levels of eGFR (quartiles 1 to 3) in normal-to-mild reduced renal function groups. However, the OR for high predicted Framingham CHD risk was abruptly decreased in the moderate-to-severe reduced renal function group (quartile 4). The results indicated that the association between high predicted Framingham CHD risk and detectable hs-cTnT gradually strengthened with decreasing levels of eGFR in normal-to-mild reduced renal function. However, this kind of association was weakened in moderate-to-severe reduced renal function. Combined with the results of table 3, one possible and reasonable explanation for the abruptly decreasing of OR was that eGFR had an effect on detectable hs-cTnT in moderate-to-severe reduced renal function. In other words, we thought that the high predicted Framingham CHD risk was independently associated with detectable hs-cTnT in all levels of eGFR, while reduced level of eGFR as an independent factor was added on high predicted Framingham CHD risk in association with detectable hs-cTnT in moderate-to-severe reduced renal function.

Our study was a cross-sectional survey and, as such, did not have the ability to determine the causes of hs-cTnT elevation in the context of impaired renal function. But our results suggest that sub-clinical myocardial injury due to cardiovascular risk factors (indicated by the high predicted Framingham CHD risk) maybe play a dominant role in hs-cTnT elevation. Our data also suggest that reduced hs-cTnT clearance was not independently associated with hs-cTnT elevations in subjects with normal or mildly reduced renal function. However, in the context of severely impaired renal function, both increased sub-clinical myocardial injury and reduced hs-cTnT clearance may be responsible for elevated hs-cTnT levels. This is a complex issue and further studies are needed to clarify these relationships.

In the present study, GFR was estimated by modifying the MDRD equation based on data from a Chinese population. It offered significant advantages in different stages of chronic kidney disease (CKD). In particular, underestimation of GFR in CKD stages 1–2 was significantly improved, resulting in a lower overestimation of prevalence of reduced renal function if used in a Chinese population [19]. By using this equation, the prevalence of moderate-to-severe reduced renal function in our study was estimated to be 1.89%. This value was similar to the result from another community-based study in China in which the prevalence of decreased renal function was 1.7% [38].

The small number of the community-based population was limitation in the present study and these participants may not be fully representative of the general population. Moreover, the study population was Chinese and renal function was estimated by C-MDRD. Therefore, extrapolation of our results to other demographic groups should be done with caution. This study was a cross-sectional survey and had no capability to get the cause and effect, so more exact conclusions should be confirmed by a prospective study.

In conclusion, hs-cTnT levels are detectable with a highly sensitive assay in a community-based Chinese population. Renal function is associated with hs-cTnT levels. eGFR levels and the high predicted Framingham CHD risk are conjunctly associated with detectable hs-cTnT in moderate-to-severe reduced renal function. Further, large and well-conducted studies are urgently required to provide more definitive evidence.
